# Perioperative intraperitoneal chemotherapy for peritoneal surface malignancy

**DOI:** 10.1186/1479-5876-4-17

**Published:** 2006-04-10

**Authors:** Tristan D Yan, Oswald A Stuart, Dal Yoo, Paul H Sugarbaker

**Affiliations:** 1 Peritoneal Surface Malignancy Program, Washington Cancer Institute, Washington DC, USA

## Abstract

The treatment of peritoneal surface malignancy mainly focuses on diffuse malignant peritoneal mesothelioma, pseudomyxoma peritonei from appendiceal cancer, and peritoneal dissemination from gastrointestinal and ovarian cancers. Cancer progression causes peritoneal implants to be distributed throughout the abdominopelvic cavity. These nodules plus the ascitic fluid result in abdominal distension. As the disease progresses, these tumors cause intestinal obstruction leading to debilitating symptoms and a greatly impaired quality of life. In the past, the prognosis of patients with peritoneal surface malignancy was regarded dismal and cure was not an option. Recently, cytoreductive surgery combined with perioperative intraperitoneal chemotherapy has shown an improved survival in selected patients with this disease. To date, multiple different treatment regimens of perioperative intraperitoneal chemotherapy have been used. This review focuses on the perioperative intraperitoneal chemotherapy currently in use in conjunction with cytoreductive surgery for the treatment of peritoneal surface malignancy at the Washington Cancer Institute.

## Introduction

In the past peritoneal surface malignancy was considered an incurable disease. Neither systemic chemotherapy nor intraperitoneal chemotherapy alone had any significant impact on survival. Palliative debulking surgery was almost always associated with disease recurrence within a few months. As the number of repeated debulking procedures increased, patients were more likely to suffer from intestinal obstruction and fistula formation. Eventually failure of the treatments would lead to death. Recently there have been an increasing number of publications describing a comprehensive treatment approach that involves cytoreductive surgery and perioperative intraperitoneal chemotherapy. [1-15].

Cytoreductive surgery consists of a series of peritonectomy procedures and visceral resections aiming to maximally eradicate visible tumor nodules on the parietal and visceral peritoneum. This is followed by perioperative intraperitoneal chemotherapy, immediately after the cytoreductive surgery and during the early postoperative period. Perioperative intraperitoneal chemotherapy targets specifically the microscopic residual disease in an attempt to prolong disease-free survival and result in cure for selected patients. This combined surgical and chemotherapeutic cytoreductive approach has demonstrated an improved survival in selected patients with diffuse malignant peritoneal mesothelioma (DMPM), pseudomyxoma peritonei (PMP), and peritoneal dissemination from gastrointestinal and ovarian malignancies. [1-15].

However, the treatment protocols of perioperative intraperitoneal chemotherapy vary from one institution to another and there has been no real consensus on the most effective regimens that should be used. This review focuses on the pharmacologic information available for the intraperitoneal chemotherapeutic agents routinely used for peritoneal surface malignancy at the Washington Cancer Institute, Washington DC.

### Properties of Perioperative intraperitoneal chemotherapy

Drugs selected for intraperitoneal administration usually are hydrophilic and have large molecular size, so that they pass slowly through a peritoneal-plasma barrier and are therefore more effectively sequestered in the peritoneal cavity [16]. The area under the curve ratio of intraperitoneal concentration to plasma concentration times time for these drugs, indicates the amount of chemotherapy agent that is sequestered in the peritoneal cavity to how much of it is absorbed into the systemic circulation. A large peritoneal to plasma ratio is an important property of intraperitoneal chemotherapy, because it maintains the maximal concentration of the drug in the peritoneal cavity during the chemotherapy washing, while not necessarily increasing the systemic toxicities.

There are two time periods that have been utilized for perioperative intraperitoneal chemotherapy administration, during the operation immediately after cytoreductive surgery and during the early postoperative period. The drugs selected for intraoperative use must have some common properties [17,18]. First, they should be augmented by heat, because heat has been found to potentiate the cytotoxic effects of certain drug; also heat increases the penetration of these drugs into tumor nodules and heat itself exerts stress on tumor cells [19,20]. Second, the drugs must have rapid cytotoxic effects on tumors during the intraoperative drug administration period. The cytotoxicity should not be dependent upon cell division.

There are several different nomenclatures used for heated intraoperative intraperitoneal chemotherapy (HIIC), such as intraperitoneal hyperthermic chemoperfusion (IPHC) and hyperthermic intraperitoneal chemotherapy (HIPEC). In this review IPHC is used.

The drugs selected for administration in the early postoperative period require cell contact for a longer time periods to cause cytotoxic effects. Usually their activity is dependent on cell division. At the Washington Cancer Institute, the chemotherapy solution dwells for 23 hours and then drains for one hour prior to the next instillation. The instillation is repeated for the first 5 postoperative days [21]. During this time period a minimal postoperative adhesion process occurs, allowing a relatively uniform drug distribution of the intraperitoneal chemotherapy.

Some institutions use IPHC only; some use early postoperative intraperitoneal chemotherapy (EPIC) only and others use a combination of IPHC and EPIC.

### Diffuse Malignant Peritoneal Mesothelioma (DMPM)

#### Natural history of DMPM

DMPM arises from the serosal lining of the abdominopelvic cavity, involving the parietal and visceral peritoneum. It is characterized macroscopically by thousands of whitish tumor nodules of variable size and consistency that may coalesce to form plaques or masses or layer out to uniformly cover the entire peritoneal surface [22]. DMPM is commonly associated with debilitating ascites. In the majority of patients the disease remains localized within the abdominal and pelvic cavities throughout its course. In contrast to mucinous carcinomatosis from appendiceal cancer, the pattern of distribution of the peritoneal nodules of DMPM does not spare mobile visceral surfaces of the small bowel and its mesentery [22].

#### Intraperitoneal hyperthermic chemoperfusion for DMPM

After cytoreductive surgery, IPHC with doxorubicin (15 mg/m^2^) and cisplatin (50 mg/m^2^) is administered at approximately 42°C in 1.5% dextrose peritoneal dialysis solution for 90 minutes [11]. There is no pharmacological incompatibility between these two drugs. Doxorubicin is an antitumor antibiotic, which can be augmented by heat, with an area under the curve ratio of 230 and a penetration of at least five cell layers [19]. It has a mild sclerosing effect on the peritoneum when used at this standard dose. This sclerotic effect is potentially effective in the management of debilitating ascites in DMPM patients. [23-25].

Another favorable pharmacologic property of doxorubicin is its ability to penetrate into tumor nodules. A recent study demonstrated that the concentration of doxorubicin in the tumor nodules harvested during the intraoperative chemotherapy instillation is even higher than the concentration of doxorubicin in the intraperitoneal fluid [26]. This suggests that there may be an active uptake of doxorubicin by mesothelioma tumor nodules. A maximal chemotherapy response rate is therefore expected.

Cisplatin is an alkylating agent, with an area under the curve ratio of 10 and the ability to penetrate tumor nodules up to 3 mm at 41.5°C. The synergistic effect with heat has been considered to be a result of higher and more selective uptake of the drug by cancer cells [27]. It has been used as an intraperitoneal drug for DMPM, gastric cancer and ovarian cancer. Cisplatin is compatible with multiple other agents, such as mitomycin C, doxorubicin and etoposide thus constituting a logical component of a combination intraperitoneal chemotherapy regimen.

#### Early postoperative intraperitoneal chemotherapy for DMPM

EPIC with paclitaxel is used as the standard treatment for patients with DMPM [11]. Paclitaxel is an antimitotic drug that stabilizes microtubules and inhibits their depolymerization for free tubulin. Its area under the curve ratio is 1000 and can potentially penetrate up to 80 cell layers [28]. These properties provide great advantage for the intraperitoneal use of this drug. When paclitaxel is used in hetastarch carrier solution, the artificial ascites created exposes peritoneal surfaces to paclitaxel throughout each 23-hour dwell [29]. The hetastarch maintains an increased volume of chemotherapy solution in the peritoneal space as compared to a saline or dextrose solution, with an increased surface exposure of the abdomen and pelvis.

Our most recent update of 100 patients, who underwent cytoreductive surgery and IPHC with cisplatin and doxorubicin followed by EPIC with paclitaxel for DMPM, showed that the median survival was 50 months and the 5-year survival was 44% [30].

### Pseudomyxoma Peritonei (PMP)

#### Natural history of PMP

PMP is characterized by a copious mucin production by adenomatous tumor cells. The neoplastic cells and mucous ascites occupy predictable anatomic sites within the peritoneal cavity. PMP is the paradigm for successful treatment of peritoneal surface malignancy [4]. It is a minimally aggressive epithelial tumor, which has limited capability of invading the peritoneum.

Appendiceal tumor initially obstructs the narrow lumen of the appendix. This leads to appendiceal perforation, which allows the tumor cells to access to the peritoneal cavity. The adenomatous epithelial cells together with a large volume of mucin accumulate at the sites of peritoneal fluid absorption, such as the undersurface of the right hemidiaphragm, the greater omentum, and within dependent areas, such as pelvis, right retrohepatic space, the left abdominal gutter and the ligament of Treitz [31]. The continuous peristaltic motion of the small intestine prevents tumor implantation on its surfaces, which makes cytoreductive procedure with preservation of adequate small intestine feasible [4]. In contrast, the stomach and the large bowel are invariably involved, but to a lesser extent than quiescent surfaces such as liver and parietal peritoneum.

Mucinous appendiceal tumors distribute intraperitoneally; only rarely do they metastasize to lymph nodes or liver. The seeding of the peritoneum by mucinous tumors happens before lymph nodal or hepatic involvement [4]. After a complete cytoreduction, intraperitoneal chemotherapy can eradicate the microscopic residual disease. Consequently tumor eradication from the peritoneal space is usually possible and disease progression at systematic sites does not occur.

#### Intraperitoneal hyperthermic chemoperfusion for PMP

IPHC with mitomycin C is used for all patients with PMP. It is also used in patients with peritoneal carcinomatosis from other gastrointestinal cancers. Mitomycin C is an antitumor antibiotic, with approximately 90% of the drugs absorbed within the 90-minute intraperitoneal instillation. The area under the curve ratio is approximately 30 and the drug can potentially penetrate up to 6 cell layers. The low plasma level when administered intraperitoneally reduces the risks of bone marrow toxicities and hemolytic uremic syndrome [32]. However, even at low plasma level, mitomycin C may cause renal damage. Therefore it is important to provide forced diuresis intraoperatively. A recent pharmacokinetic study on mitomycin C dosimetry and toxicity demonstrated that volume of chemotherapy solution was very important in determining drug absorption [33]. Currently all patients with PMP receive 15 mg/m^2 ^of mitomycin C in 1.5 L/m^2 ^carrier solution at the Washington Cancer Institute.

#### Early postoperative intraperitoneal chemotherapy for PMP

5-fluorouracil is used for EPIC for peritoneal carcinomatosis from appendiceal and other gastrointestinal malignancies. This antimetabolite drug incorporates into the DNA causing chain termination. The area under the curve ratio is 250. Little 5-FU escapes into the systemic circulation, as it is metabolized by single pass through the liver via portal circulation. However, in patients with hepatic dysfunction or when co-administered with mitomycin C, a reduced dose of intraperitoneal 5-FU should be given.

A recent update on 803 patients with appendiceal epithelial neoplasms with peritoneal dissemination who were treated with cytoreductive surgery and IPHC with mitomycin C followed by EPIC with 5-FU over the last 20 years showed that the overall 5-, 10-, and 20-year survival was 63%, 54% and 50%, respectively [34].

### Colorectal Peritoneal Carcinomatosis (CRPC)

#### Natural history of CRPC

Colorectal cancer is the second most common cause of cancer-related deaths in western countries. Peritoneal dissemination is present in 10% of patients at the time of diagnosis [21]. The median survival of patients with CRPC published by Chu et al and Sadeghi et al was 6 months and 5.2 months, respectively [35,36]. Newer chemotherapeutic regimens have shown an improved response rate, but the long-term prognosis remains uniformly poor. [37-41].

#### Perioperative intraperitoneal chemotherapy for CRPC

IPHC with mitomycin C, cisplatin and doxorubicin or oxaliplatin combined with EPIC with 5-FU have been used for gastrointestinal peritoneal carcinomatosis. Oxaliplatin has been used intraperitoneally in Europe for colorectal peritoneal carcinomatosis, as pioneered by Elias and colleagues [42,43]. IPHC with oxaliplatin has a very low area under the curve ratio, which means that this drug is rapidly absorbed and does not require a long dwell time in the peritoneal cavity for maximal local-regional effect [42]. Elias and co-workers suggested that intraperitoneal oxaliplatin should be combined with intravenous 5-FU as just before IPHC [43]. This bi-directional use of intravenous and intraperitoneal chemotherapy theoretically exerts greater cytotoxic effects via both capillary network and passive diffusion. Elias et al showed that intravenous 5-FU at 400 mg/m^2 ^combined with IPHC with 460 mg/m^2 ^of oxaliplatin in 2 L/m^2 ^of peritoneal solution over 30 minutes, achieved a median survival of 60 months and a 5-year survival of 49% [43]. All patients had an optimal cytoreduction prior to the IPHC.

We participated in a recent international registry study of 506 patients with colorectal carcinomatosis treated by cytoreduction and perioperative intraperitoneal chemotherapy with a curative intent from 28 institutions. This study demonstrated that the overall median survival was 19.2 months [15]. Our recent update showed 3- and 5-year survival of 44% and 32% in 70 patients who underwent complete cytoreduction and PIC for CRPC [44].

### Gastric Peritoneal Carcinomatosis (GPC)

#### Natural history of GPC

The annual incidence of gastric cancer in the United States is 22,400 and approximately 14,400 patients die of the disease. At the time of surgery 20–30% of patients with gastric cancer being explored for potentially curative resection will be found to have peritoneal seeding [24,25,45]. Forty to fifty percent of patients who had undergone curative resection developed locoregional recurrence one to three years after their initial surgery [45]. Even at death, the tumor often remains confined to the abdomen [45].

The tumor cell entrapment hypothesis suggests that surgical manipulation of the cancer-bearing organs, transection of lymphatic channels and blood loss from the cancer specimen results in free intraperitoneal cancer cells. These cancer cells are embedded in fibrin, as the wound-healing process is initiated [45].

Current standard treatment is systemic chemotherapy for patients with GPC, which may delay onset of symptoms, but does not have any significant impact on survival. The high incidence of isolated peritoneal carcinomatosis from gastric cancer suggests that cytoreductive surgery and perioperative intraperitoneal chemotherapy in selected patients could result in improved survival.

#### Perioperative intraperitoneal chemotherapy for GPC

Cytoreductive surgery is combined with hyperthermic intraperitoneal perfusion with mitomycin C, cisplatin and etoposide [46]. A recent study reported to use neoadjuvant intraperitoneal chemotherapy with docetaxel and carboplatin combined with intravenous methotrexate and 5-FU for advanced GPC [47].

### Ovarian Peritoneal Carcinomatosis (OPC)

#### Natural history of OPC

Epithelial ovarian cancer accounts for 80–90% of all ovarian malignancies and is the main cause of death for all gynecological tumors [48]. Tumor dissemination is frequently confined to the peritoneal cavity. Papillary serous carcinoma of the peritoneum is an extra-ovarian primary peritoneal carcinoma. The epithelial layer of the ovary and the peritoneum have a common origin from the coelomic epithelium. Cytoreductive surgery is considered as the standard of care for ovarian malignancies. This is usually followed with systemic chemotherapy as most patients with ovarian cancer are chemotherapy sensitive and a prolonged survival is expected [49,50]. However, disease recurrence is common and often resistant to additional treatment with systemic chemotherapy. There is a potential role for cytoreductive surgery combined with perioperative intraperitoneal chemotherapy for advanced primary and recurrent epithelial ovarian cancers and papillary serous carcinoma of the peritoneum. In our previous update of 28 female patients who underwent cytoreductive surgery and IPHC or EPIC for OPC, the median survival was 46 months [14].

#### Perioperative intraperitoneal chemotherapy for OPC

Prevention and eradication of peritoneal carcinomatosis through the use of intraperitoneal chemotherapy was declared standard of practice by the National Cancer Institute, Bethesda, USA after a recent phase III study in ovarian cancer [51]. Cyclophosphamide is the only chemotherapeutic agent approved by the U.S. Food and Drug Administration for intraperitoneal use and is very effective against ovarian cancer. However, from a pharmacologic standpoint, it needs to be activated by hepatic microsomal enzymes, which makes it theoretically ineffective for intraperitoneal use. Both ifosfamide and cyclophosphamide are markedly synergized by heat. These drugs may double their cytotoxicity for cancer cells when used with hyperthermia [52]. When used intravenously these drugs are "heat targeted" to the warm peritoneal surface resulting from IPHC. A study is underway to investigate IPHC of cisplatin and doxorubicin combined with intravenous ifosfamide after cytoreductive surgery for peritoneal dissemination from advanced primary or recurrent epithelial ovarian cancer and papillary serous carcinoma. It is hoped that this bi-directional local-regional and systemic chemotherapy treatment will result in an improved survival in these patients.

### Future directions

#### Standardization of treatment protocols

This comprehensive treatment of cytoreductive surgery combined with perioperative intraperitoneal chemotherapy has shown long-term survival in selected patients with DMPM, PMP, and peritoneal dissemination from other gastrointestinal and ovarian cancers. However, there has been little uniformity in the surgical approach or the chemotherapeutic regimens used. A standardization of the treatment protocols is necessary, for multi-institutional treatment plans to provide meaningful data. Some new concepts regarding neoadjuvant chemotherapy, bi-directional chemotherapy, and adjuvant chemotherapy for treatment and prevention of disease recurrence are theoretically appealing.

#### Neoadjuvant chemotherapy

Yonemura and co-workers used neoadjuvant intraperitoneal and systemic (NIPS) chemotherapy in patients with GPC to downstage the tumor volume [47]. After NIPS they achieved complete tumor resection in 25% of patients [47]. Theoretically, this chemoselection approach can also be used in patients with CRPC. The prognosis in both diseases is heavily dependent on the tumor volume at the time of surgical exploration.

#### Bi-directional chemotherapy

The extent of intraperitoneal chemotherapy penetrating tumor nodules by passive diffusion is limited to a few cell layers. Elias and colleagues have been using hyperthermic intravenous chemotherapy in combination with intraperitoneal chemotherapy to achieve a greater response. The cytotoxic effects result not only from penetration within the tumor via the capillary network but also by passive diffusion from the peritoneal space. In a recent study they have reported a 5-year survival rate of 49% in 30 patients who underwent complete cytoreduction for CRPC [43]. However, it remains unclear in terms of how much of the improved survival is contributed by the bi-directional perioperative chemotherapy treatment and how much of it is due to the patient selection. All their patients treated with perioperative chemotherapy had a complete cytoreduction. It would be interesting to see if this bi-directional chemotherapy approach can pharmacologically improve chemotherapy penetration and more importantly if this approach can broaden the scope of patients who can potentially benefit from cytoreductive surgery and perioperative intraperitoneal chemotherapy.

#### Adjuvant intraperitoneal chemotherapy

The peritoneal surface is a frequent site of tumor recurrence after primary cancer surgery. This occurs because free cancer cells are shed from the primary tumor or present in blood and lymph in the surgical field. In some patients it is impossible to avoid dissemination of cancer cells during surgical manipulation. Yu and co-workers reported an improved survival in patients who received adjuvant intraperitoneal chemotherapy for advanced gastric cancer [53]. Xu and colleagues have recently suggested in a meta-analysis an improved management of advanced gastric cancer when IPHC was used in conjunction with complete resection (Hazard ratio: 0.51 and 95% confidence interval: 0.40 – 0.65) [54]. In 8 of the 11 studies, mitomycin C was used.

When all the data is considered together it may not be unreasonable to use perioperative chemotherapy in all gastrointestinal patients who are at higher risk of developing local recurrence or peritoneal dissemination. These are the patients who have full thickness cancer involvement of the lumenal wall, bowel perforation, localized peritoneal implants, Krukenberg's tumors and in patients where an *en-bloc *removal of tumor is not achieved.

In summary, this review focused on the perioperative intraperitoneal chemotherapy currently used for the treatment of peritoneal surface malignancy at the Washington Cancer Institute. There is a need to standardize the treatment regimens and consider incorporating perioperative intraperitoneal chemotherapy not only in the management of biopsy proven carcinomatosis, but also in the treatment of primary disease.
